# Genotypic Diversity and Short-term Response to Shading Stress in a Threatened Seagrass: Does Low Diversity Mean Low Resilience?

**DOI:** 10.3389/fpls.2017.01417

**Published:** 2017-08-14

**Authors:** Suzanna M. Evans, Adriana Vergés, Alistair G. B. Poore

**Affiliations:** ^1^Evolution and Ecology Research Centre, School of Biological, Earth and Environmental Sciences, University of New South Wales, Sydney NSW, Australia; ^2^Centre for Marine Bio-Innovation, School of Biological, Earth and Environmental Sciences, University of New South Wales, Sydney NSW, Australia; ^3^Sydney Institute of Marine Science, Mosman NSW, Australia

**Keywords:** seagrass, *Posidonia*, clonality, genotypic diversity, stress tolerance, shading, photosynthesis, resilience

## Abstract

Seagrasses that are predominantly clonal often have low levels of genetic variation within populations and predicting their response to changing conditions requires an understanding of whether genetic variation confers increased resistance to environmental stressors. A higher level of genetic diversity is assumed to benefit threatened species due to the increased likelihood of those populations having genotypes that can persist under environmental change. To test this idea, we conducted an *in situ* shading experiment with six geographically distinct meadows of the threatened seagrass *Posidonia australis* that vary in genetic diversity. Different genotypes within meadows varied widely in their physiological and growth responses to reduced light during a simulated short-term turbidity event. The majority of meadows were resistant to the sudden reduction in light availability, but a small subset of meadows with low genotypic diversity were particularly vulnerable to the early effects of shading, showing substantially reduced growth rates after only 3 weeks. Using the photosynthetic performance (maximum quantum yield) of known genotypes, we simulated meadows of varying genetic diversity to show that higher diversity can increase meadow resilience to stress by ensuring a high probability of including a high-performing genotype. These results support the hypothesis that complementarity among genotypes enhances the adaptive capacity of a population, and have significant implications for the conservation of declining *P. australis* meadows close to the species range edge on the east coast of Australia, where the genotypic diversity is low.

## Introduction

In the same way that species diversity can positively influence ecosystem processes and functions, genetic diversity within species can also improve the stability and functioning of populations, particularly during stressful events (Reusch et al., 2005; Hughes et al., 2008; Salo and Gustafsson, 2016). Increasing the number of genotypes within a population can enhance productivity (Crutsinger et al., 2006; Aguirre and Marshall, 2012) and increase resilience (Massa et al., 2013), with benefits that cascade to the wider ecosystem (Crawford and Rudgers, 2013). It is therefore expected that a population with a wider range of genotypes and corresponding phenotypes will have better ‘insurance’ against the effects of environmental stress (Foster Huenneke, 1991).

In relatively stable environments where biotic and/or abiotic conditions remain constant, differences in the success of particular genotypes can lead to the dominance of one or a few clones, and thus an overall decrease in the number of genotypes over time (McLellan et al., 1997). Should environmental conditions change, however, low diversity populations are considered at a greater risk of extinction compared to genetically diverse populations. Genotypes that are well-adapted to local conditions will not necessarily perform well in altered conditions. When genotypic diversity is high, it is statistically more likely that one or more individuals will have a suitable genotype that will thrive under the new conditions. The mechanism by which this occurs is known as complementarity, whereby the inclusion of a variety of genotypes, and thus phenotypes, allows the population access to different pools of resources, limiting competition between individuals while at the same time improving the likelihood of population success under changing conditions (Hughes et al., 2008). Ultimately, increasing the pool of genetic diversity among clonal genotypes improves evolutionary potential and adaptive capacity, particularly when faced with environmental disturbance.

Organisms that utilize predominantly clonal growth strategies are usually expected to have naturally low levels of genetic variation (Ellstrand and Roose, 1987, although there are exceptions, e.g., Sánchez-Vilas et al., 2010) and the responses of individuals to stress, including rapidly changing conditions, often involves phenotypic plasticity – the ability of an organism to modify its phenotype in response to their environment (Nicotra et al., 2010). The expression of phenotypic plasticity can vary among genotypes (Bradshaw, 2006; Lande, 2009). For example, a plastic response to environmental change can be as simple as stunted growth when nutrients are lacking, but the degree to which growth is limited by nutrients may differ among genotypes, representing genetic differences in their tolerance to stress (Schlichting, 1989). Predicting the responses of populations to environmental stress thus requires an understanding of the genetic variation in stress tolerance (Munday et al., 2013), commonly assessed by experimental designs in which the performance of individual genotypes is quantified across environments (e.g., Clark et al., 2013; Foo et al., 2014). If genotypes vary in their plastic responses (indicated by a genotype by environment interaction) then greater genetic diversity should lead to a higher likelihood of populations including tolerant genotypes.

Some of the most compelling demonstrations that genotypic diversity influences ecosystem functioning have come from experimental manipulations of diversity in seagrass ecosystems (Procaccini et al., 2007). Seagrasses provide an ideal model ecosystem to test ideas of genotypic diversity because they reproduce both sexually through seed and pollen dispersal and clonally via the vegetative growth of rhizomes, generating variation in genetic diversity within and among meadows (e.g., Ort et al., 2012; Evans et al., 2014). Over the past decade, experiments have shown that increasing the number of genotypes within an experimental plot can increase resistance to grazing (Hughes and Stachowicz, 2004), influence grazer biomass (Hughes et al., 2010), enhance resistance and resilience to invasion (Massa et al., 2013), and result in greater shoot densities and biomass compared to monocultures during disturbance events (Reusch et al., 2005; Hughes and Stachowicz, 2011). There is also evidence to suggest that rehabilitation and restoration efforts can be significantly enhanced by including a range of different genotypes sourced from multiple donor locations (Procaccini and Piazzi, 2001; Williams, 2001; Reynolds et al., 2012).

The relationship between genotypic diversity and resilience in seagrass meadows is particularly important in plant responses to extreme events, such as heat waves (Reusch et al., 2005), unusually intense grazing (Hughes and Stachowicz, 2004) and eutrophication (Arnaud-Haond et al., 2010). Less well understood is whether genotypic diversity alone can buffer against the effects of more typical, short-term environmental stresses that are not necessarily extreme and do not always result in high shoot mortality (Salo et al., 2015).

In this study, we examined how individual genotypes vary in their response to short-term stress in the threatened seagrass *Posidonia australis*. We use this predominantly clonal species to contrast how individual genotypes, and meadows of varying genetic diversity, perform under a simulated stress due to increased shading. Reduced light availability due to turbidity is one of the most common stressors facing seagrass meadows globally (Waycott et al., 2009). Rapid increases in turbidity are often caused by anthropogenic inputs to coastal waters, particularly via fine sediment re-suspension, nutrient over-enrichment and pollution (Ralph et al., 2006). Such turbidity increases are most often derived from industrial and agricultural run-off into estuaries and bays, commercial dredging, coastal construction, stormwater, and sewage (Pergent-Martini and Pergent, 1996; Short et al., 1996).

By utilizing carbohydrates stored within the rhizome, seagrasses can endure short periods of light reduction (for example during ‘pulsed’ turbidity events such as those following storms or heavy rain; (Longstaff and Dennison, 1999). However, extended periods of shading can severely impair growth rates and productivity (e.g., Collier et al., 2012), resulting in short, stunted growth forms (Walker et al., 1999), reduced shoot densities and biomass, and ultimately widespread decline (Short and Wyllie-Echeverria, 1996).

The genotypic diversity of *P. australis*, as measured using microsatellite DNA markers, varies greatly among meadows in south-eastern Australia (Evans et al., 2014), and this diversity reflects phenotypic variation in functionally relevant traits such as leaf morphology and productivity (Evans et al., 2016). Slow-growing and large seagrass species such as *P. australis* and *P. oceanica* are seemingly more tolerant to extended periods of shade than faster growing species such as *Zostera marina* and *Halophila ovalis*, which generally show rapid responses to shading (reduced shoot densities, high shoot mortality, and rapid recovery; (Ochieng et al., 2010; Yaakub et al., 2014). Currently, for slow-growing species that are poor colonizers, such as *P. australis*, one of the only definitive indicators that the seagrass is under stress is meadow dieback (Gobert et al., 2006). For conservation purposes, this is obviously not an acceptable indicator of stress, as by then the plants have already reached a critical state.

Shading studies conducted in seagrass meadows often have a long duration of months to years, in which seagrass recovery following the removal of shades is also recorded (e.g., Fitzpatrick and Kirkman, 1995; Collier et al., 2009; McMahon et al., 2011, but see Salo et al., 2015). More often than not, records of *P. australis* meadows that have experienced shoot fatality/decreased density have not shown any signs of recovery (Walker and McComb, 1992; Fitzpatrick and Kirkman, 1995), or recovery has been extremely slow (Meehan and West, 2004). In the context of rehabilitation and conservation efforts, it is therefore most important to determine the susceptibility of seagrasses to shading stress *prior* to fatality, considering that shoot mortality is the endpoint we wish to avoid. Understanding what factors can predict resilience and tolerance to stress are therefore required to underpin the future monitoring and management of these meadows. Consequently, we performed a short-term shading experiment *in situ* to answer whether individual genotypes within meadows varied in their response to short-term light reduction, using leaf growth rates and photosynthetic efficiency as indicators of performance. We then used these genotype-level results compiled across six meadows to model the likelihood of including a high-performing genotype within meadows of differing genotypic diversity levels.

## Materials and Methods

### Study Species

The seagrass *Posidonia australis* is endemic to temperate Australian waters, extending from Shark Bay in Western Australia, around the southern coastline (including northern Tasmania) to Wallis Lake in New South Wales (Creese et al., 2009). The growth form of *P. australis* is predominantly clonal, particularly on the east coast of Australia, where it has relatively low genetic diversity (Evans et al., 2014) and flowering events are considered an ‘episodic phenomenon’ (Gobert et al., 2006). Genetic diversity increases with latitude, southward from the northern range edge at Wallis Lake (Evans et al., 2014). Vegetative growth is extremely slow with the rate of rhizome spread for *P. australis* estimated at 0.3–21 cm per year (Gobert et al., 2006). Successfully established recruits can take 10 or more years to mature (Meehan and West, 2004), generating clones with remarkably long lifespans of potentially hundreds to thousands of years (similar to *P. oceanica* in the Mediterranean, Arnaud-Haond et al., 2012).

### Site and Genotype Selection

**FIGURE 1:**
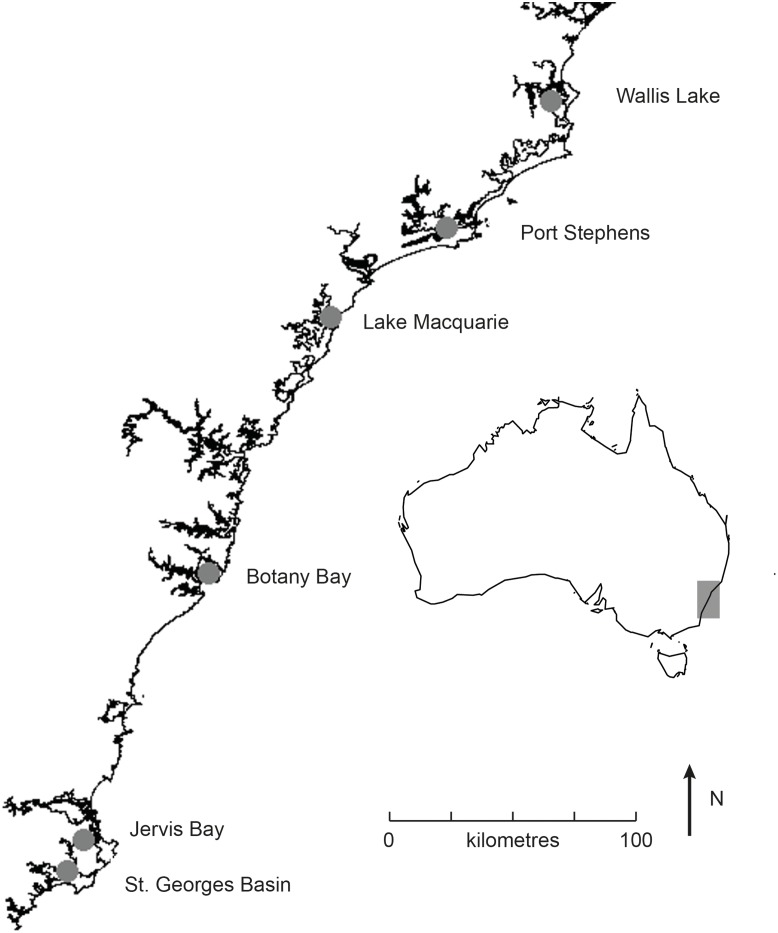
Map showing the six estuaries in which shading experiments were conducted along the east coast of Australia (region represented by gray box on inlaid map).

Six estuaries along the New South Wales coast containing *Posidonia australis* meadows were chosen for this study, with two meadows sampled within each estuary (**Figure 1**). Site choice was based on the genetic diversity in these meadows previously quantified by Evans et al. (2014). The 12 meadows of *P. australis* sampled displayed considerable variation in genetic diversity among estuaries, from low diversity meadows dominated by only one genotype (Wallis Lake, Lake Macquarie, and Jervis Bay) (Evans et al., 2014) despite the existence of between two and three genotypes within the meadows) to higher diversity meadows (Botany Bay, Port Stephens, and St. Georges Basin) with 5 to 21 genotypes per meadow, and not strongly dominated by large patches of a single clone (**Table 1**).

**Table 1:** *Posidonia australis* meadows chosen as locations for shading experiments.

Location	MLG	*R*	Ho	He
Wallis Lake	2	0.03	0.183	0.192
Lake Macquarie	3	0.07	0.379	0.191
Jervis Bay	3	0.07	0.333	0.184
Port Stephens	5	0.14	0.675	0.138
Botany Bay	7	0.21	0.092	0.144
St. Georges Basin	21	0.69	0.292	0.278


*MLG, number of multilocus genotypes; R, clonal richness; Ho, observed heterozygosity; He, expected heterozygosity (Evans et al., 2014)*.

Using information gathered from previous genetic sampling (Evans et al., 2014), we generated maps of the locations of different genotypes within each of the six meadows. At each meadow, three patches were selected (separated by at least 10 m) where it could be safely assumed that different genets would be sampled, with the exception of Wallis Lake, where only two genotypes were found.

All shoots were sampled at a similar depth of approximately 1.5 m, to avoid potential differences in light-acclimation with depth (known for *Posidonia oceanica*, Dattolo et al., 2017). From each of these patches, an individual ramet was collected with at least nine shoots connected by the same rhizome (and thus the same genetic clone). The rhizome was then cut between each shoot, thus leaving nine separate shoots with the same genotype. The effects of this manipulation were tested in a pilot study (details below). Each shoot was left with at least 5–10 cm of rhizome attached. Using this method, 27 shoots (consisting of three genotypes) were harvested at each location. Each individual shoot was then assigned to one of three treatments: shaded, mesh, or control. Each treatment was replicated three times, with each individual shoot being assigned to each treatment (i.e., three separate shoots under three separate shaded areas). To distinguish among genotypes, one of three colors (pink, orange, or green) were assigned to each genotype, with that color flagging tape then attached adjacent to the corresponding treatment. Shoots were re-planted into bare sediment directly adjacent to surrounding patches and anchored with a tent peg.

### Shading Treatment

Shades were created using gardening shade-cloth (70% UV block) that was then folded in half to create a double layer and cut into 50 cm × 50 cm squares. Four lengths of rope (30 cm each) were attached to each corner, with the other end firmly affixed to a tent peg. The tent pegs served as anchors for the shade cloth, which floated 30 cm above the sediment without touching the seagrass canopy. A small (5 cm diameter) polystyrene ball was attached to each corner of the shade cloth to keep the shade floating above the seagrass at all times. The lengths of rope attached to the shade and anchored into the sediment allowed natural water flow to continue, without disrupting the shade cloth or restricting water movement around the seagrass.

To ensure that the physical presence of the shade cloth was not a confounding artifact, procedural controls were created in the same manner as the shading treatments using a black plastic 2 cm garden mesh in place of the shade cloth. Analyses of variance (ANOVA) revealed no significant differences between the procedural controls and the un-manipulated controls for growth (*F*_1,83_ = 0.46, *P* = 0.53) or fluorometry measurements (*F*_1,83_ = 0.01, *P* = 0.93) and, thus, the data from the procedural controls were not included in further analyses. Un-manipulated controls consisted of no shading or mesh, with only a tagged bamboo stick pushed into the sediment adjacent to the seagrass shoot. To test the efficacy of the shades in reducing available light, we used a diving-PAM (Walz, Germany) with an external fiber quantum sensor to record photosynthetically active radiation (PAR; μmol m^-2^ s^-1^) directly beneath the shaded and mesh treatments, and adjacent to unshaded controls. These values were then compared using a one-way ANOVA.

### Growth Rates

Leaf growth rates were measured as an estimate of shoot productivity. To do this, hypodermic needles were used to punch three parallel holes just above the ligule of each shoot, penetrating all leaves (Romero, 1989). As leaves grow from the ligule, we could then estimate leaf growth after harvesting by measuring the displacement of the holes relative to the ligule of the oldest non-growing leaf. These values were then converted to production of new biomass in dry weight per shoot per day (mg dw shoot^-1^ day^-1^) by drying all leaves to a constant weight at 60°C for 48 h. Leaf turnover in *Posidonia australis* takes approximately 54 days (Short and Duarte, 2001); in this experiment shoots were harvested after 21 days, ensuring that the leaf scars would still be present.

Seagrasses are known to translocate resources (including photosynthates) between shoots of the same rhizome (Marbà et al., 2006). This can lead to shading studies becoming confounded, as shaded shoots can continue to thrive by accepting translocated photosynthates from nearby unshaded shoots. To avoid this and to ensure that the growth of a given replicate shoot was not influenced by neighboring shoots, rhizomes were cut at the edges of the plots within the shade treatments to physically separate the experimental shoot from any neighboring shoots. To test whether separating individual shoots at the rhizome would impact leaf growth rates over the short time frame of the experiment (3 weeks), growth rates were measured *in situ* using five shoots cut at the rhizome and replanted (anchored with a tent peg) and five un-manipulated shoots. Three parallel holes were made at the ligule of each shoot to measure growth as per the above methods. These measurements were compared between treatments after 3 weeks using a *t*-test. All shoots survived, and there was no difference in the growth of separated vs. undisturbed shoots (*t* = 0.82, df = 8, *P* = 0.41).

Analyses of variance were conducted on the growth data using treatment (shaded or control) as a fixed factor, meadow as a random factor and genotype as a random factor (nested within meadow). Tukey’s *post hoc* analyses were used to further determine differences between meadows. The data were log (*x* + 1) transformed prior to analyses.

### Fluorometry

Pulse-amplitude modulated (PAM) fluorometry was used to estimate the photosynthetic efficiency of each shoot. This was done using a mini-PAM (Walz, Germany) immediately after the shoots were harvested. Shoots from shaded treatments were collected in canvas bags to ensure the effects of shading on photophysiology were not reversed before fluorometry could take place. All shoots were dark adapted using leaf clips for 10 min prior to measurements. All fluorometry measurements took place during June 2013, between 10:00 and 14:00 h during low tide, so that data could be gathered about the performance of the species under the high irradiance conditions it naturally experiences, and so that accurate comparisons could be made between sites.

Exposure to very high irradiance can induce a decline in photosynthetic activity, termed photoinhibition, which results in a loss of variable chlorophyll fluorescence (Beer et al., 2014). One of the most frequently used parameters in chlorophyll fluorescence is the ratio between variable and maximum fluorescence, known as maximum quantum yield, or *F*_V_/*F*_M_. This is a measure of photochemical efficiency, which is useful for understanding the physiological state of photosystem II (PSII), especially when plants are under stress (Van Goethem et al., 2013).

Values of *F*_V_/*F*_M_ are strongly influenced by very recent local light conditions. Consequently, it is not appropriate to compare differences in this trait on control plants from different estuaries, as values will naturally vary widely depending on factors such as specific time of day, cloud cover, tidal height, etc. For this reason, differences in *F*_V_/*F*_M_ between genotypes and meadows were quantified using shaded treatments only, as these all shared similar reduced incident light conditions equivalent to less than 5% of surface irradiance. Differences in *F*_V_/*F*_M_ of the shaded plants were quantified using ANOVA with meadow as a random factor and genotypes as a random factor nested within meadow.

Generally, photosynthetic efficiency is considered ‘optimal’ when *F*_V_/*F*_M_ averages 0.83 (the global terrestrial constant reported in the literature; Björkman and Demmig, 1987). We thus used an *F*_V_/*F*_M_ value of 0.8 as a threshold to determine whether individual shoots were considered high-performers under reduced light conditions. In order to calculate the likelihood that a meadow with a given genotypic richness (e.g., 2, 5, 10 genotypes) will possess a high performing genotype (*F*_V_/*F*_M_ values of > 0.8), we pooled all available *F*_V_/*F*_M_ data and repeatedly resampled the data 1000 times with varying numbers of genotypes in the sample (from 2 to 17). For each sample, records were made of any genotypes with *F*_V_/*F*_M_ values greater than 0.8. The resulting proportions were then plotted against genotypic richness.

## Results

The use of shade cloth significantly reduced the levels of photosynthetically active radiation (PAR, 400–700 nm) in contrast to the mesh and unshaded control treatments (*F*_2,24_ = 8.93, *P* = 0.001). PAR under the shades averaged 20.2 ± 2.4 μmol photons m^-2^ s^-1^, compared to PAR adjacent to controls, which averaged 237 ± 19.7 μmol photons m^-2^ s^-1^. The reduction in light created by the shaded treatments was comparable to that caused by ‘high’ natural turbidity levels in coastal benthic habitats (Longstaff and Dennison, 1999).

**FIGURE 2:**
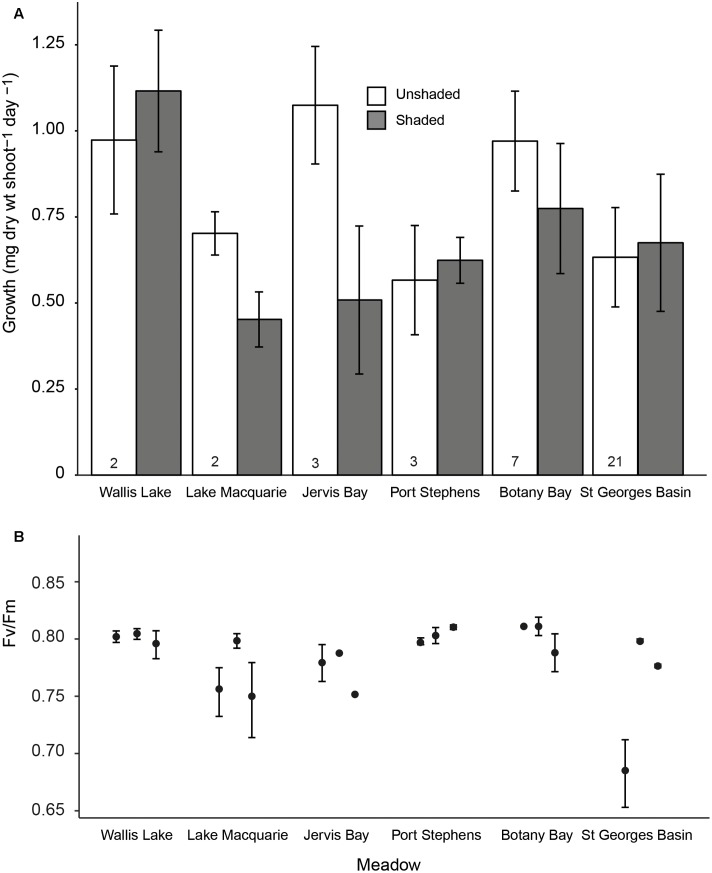
**(A)** Mean leaf growth rates (mg dw shoot^-1^ day^-1^ ± SE) for shaded and control treatments in each of six meadows. The numbers at the base of each bar are the genotypic richness of each meadow; and **(B)** mean maximum quantum yield (*F*_V_/*F*_M_ ± SE) for each of three genotypes within each meadow following 3 weeks of shading (note the *y*-axis starts at 0.65).

**FIGURE 3:**
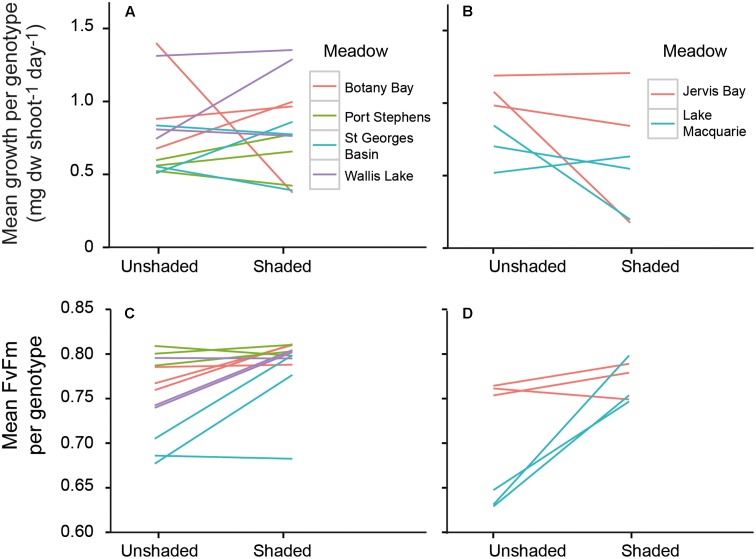
Reaction norms of individual genotypes across shaded and control treatments for growth rates (top panel) and photosynthetic efficiency (bottom panel) of genotypes from Wallis Lake, Port Stephens, Botany Bay, and St. Georges Basin **(A,C)**, and Lake Macquarie (dark gray lines) and Jervis Bay (dashed lines) **(B,D)**. Lines join the mean response for each genotype across the two light environments.

The growth rates of *P. australis* varied among meadows, and the effects of shading varied among the meadows (**Figure 2A**, significant meadow × treatment interaction, **Table 2**). This was due to a significant reduction in growth rates due to the shaded treatments in two meadows (Lake Macquarie and Jervis Bay; **Figure 2A**). Both of these meadows are considered to have very low genetic diversity, with only three genotypes present (Evans et al., 2014). Shading treatments did not significantly impact the growth rates of the three ‘higher’ genotypic diversity meadows (Botany Bay, Port Stephens, and St. Georges Basin), nor the meadow with the lowest genotypic diversity, Wallis Lake, which supports only two distinct genotypes (**Table 2**). The effects of shading did not vary among individual genotypes within a meadow (genotype × treatment interaction non-significant, **Table 2**), visualized by the slope and elevation of their reaction norms across treatments (**Figure 3A**).

**Table 2:** Analysis of variance for the growth rate of *P. australis* contrasting treatments (shaded or control, fixed factor), meadows (random factor), and genotypes (random factor, nested within meadow).

Source	df	MS	*F*	*P*
Meadow	5	0.50	2.30	0.12
Treatment	1	0.43	0.88	0.40
Genotype (Meadow)	12	0.22	1.19	0.31
Meadow × Treatment	5	0.50	3.45	**0.04**
Treatment × Genotype (Meadow)	12	0.15	0.78	0.65
Residual	55	0.19		


*Significant factors (P < 0.05) are shown in bold*.

**FIGURE 4:**
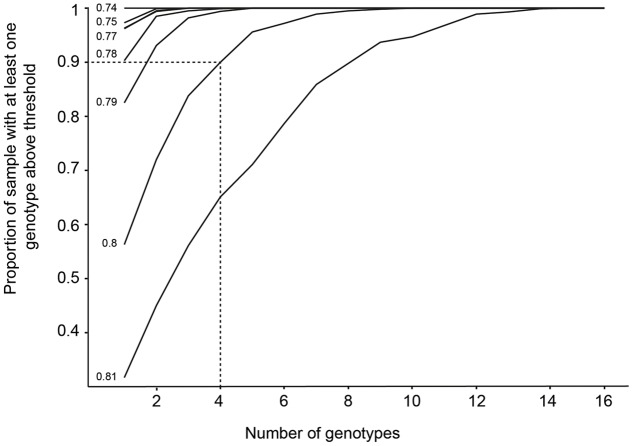
The likelihood that a sample with a given number of genotypes will possess a high performing genotype. The *y*-axis represents the likelihood of including a genotype performing at the *F*_V_/*F*_M_ values listed on the left of each line (0.74–0.81), generated by resampling the pooled original data 1000 times. For example, the dashed line illustrates a scenario, where at least four genotypes would be needed within a meadow to achieve a 90% or greater likelihood of including a high-performing genotype with a photosynthetic efficiency of 0.8.

Values of *F*_V_/*F*_M_ under the shaded treatments significantly differed among genotypes, but not among meadows (**Figures 2B, 3B** and **Table 3**). To model the effect of meadow genotypic richness on sensitivity to light reduction, we first compiled the mean values of *F*_V_/*F*_M_ for each sampled genotype across all meadows. The values of *F*_V_/*F*_M_ among shaded treatments varied between 0.68 and 0.81 and we considered plants showing *F*_V_/*F*_M_ values of 0.8 or greater to be ‘high performers,’ or the plants best acclimated to light reduction. *F*_V_/*F*_M_ responses from all individual genotypes (two or three per meadow) were then combined to create a theoretical pool of regional genotype responses. Repeated resampling of the pooled data generated the likelihood that a meadow with a given number of genotypes (from 2 to 16) would produce one or more high performers. Modeled simulations showed that for a theoretical meadow from our sampled region to have a 90% or greater likelihood of having one or more genotypes performing at ≥0.8, the presence of at least four genotypes would be required (**Figure 4**). A 90% chance of including a genotype with the higher performance of 0.81 would require approximately eight genotypes.

**Table 3:** Analysis of variance for maximum quantum yield, *F*v/*F*m, of *P. australis* in the shaded treatment contrasting meadows and genotypes within meadows.

Source	df	MS	*F*	*P*
Meadow	5	0.00314	1.78	0.16
Genotype (Meadow)	12	0.00183	2.69	**0.03**
Residual	23	0.00068		


*Significant factors (P < 0.05) are shown in bold*.

## Discussion

Our results show that the majority of *Posidonia australis* meadows are resilient to short-term shading stress. However, a subset of meadows in two low diversity estuaries were particularly vulnerable to the early effects of shading. We found that different genotypes within meadows varied in their physiological responses to shading, and estimate that approximately four or more genotypes are required to ensure a high probability of including a ‘high-performing’ (i.e., higher photosynthetic efficiency) genotype in a theoretical meadow consisting of random genotypes from the range of meadows sampled.

The vast majority of *P. australis* meadows on the east coast of Australia grow in shallow (1–5 m) waters just below the low tide mark (West, 1990) and are thus subjected to rapid changes in light from turbidity. Changes in light availability can trigger a wide range of physiological responses in seagrasses (Kumar et al., 2016) and our results suggest that in the short-term, *P. australis* meadows are generally very resilient to reduced light. For the majority of meadows, shading did not significantly impact seagrass growth rates over the course of the experiment. This contrasts with the findings of a similar study performed in *Zostera marina*, where shading over a similarly short time frame resulted in suppressed eelgrass growth rates for a range of genotypes (Salo et al., 2015). This may be explained by the fact that *Z. marina* is a faster-growing species with a smaller rhizome and reduced likelihood of translocation of stored resources.

We found that the degree to which shading affected growth varied among meadows as a result of two meadows with low genotypic richness, Lake Macquarie and Jervis Bay, both experiencing a ∼60% reduction in mean growth rates per meadow under the shaded treatments compared to controls. The growth of *P. australis* varied among genotypes, but there was no interaction between genotype and shading treatment, indicative of variation in stress tolerance among genotypes within a meadow. While these two meadows share a low genotypic richness, we cannot discount other environmental variables that were not measured and are likely to vary on large scales among all our sampling sites (e.g., salinity and nutrient supply). Lake Macquarie and Jervis Bay, however, are very different estuaries. Lake Macquarie is characterized by industrial and residential development, with a small *P. australis* meadow that was listed as threatened under the Australian Environment Protection of Biodiversity Act 1999 in 2015. Contrastingly, Jervis Bay is an open embayment, where some of the largest *P. australis* meadows on the east coast of Australia are protected within a marine park (Creese et al., 2009). This suggests that the overall vulnerability of Lake Macquarie and Jervis Bay *P. australis* meadows to short-term stress is at least partly due to the existence of particular genotypic individuals within the meadows that are less resilient to light reduction than others rather than shared environmental conditions.

The mechanisms behind which genotypic diversity can enhance performance under stress include both complementarity and dominance (Hughes and Stachowicz, 2009). For strong dominance to occur, a small number of genotypes will become very abundant within a population; a common occurrence in slow-growing, predominantly clonal seagrass meadows such as *P. australis* (Evans et al., 2014). Meadows of *P. australis* on the east coast of Australia are presumably long-established (following the stabilization of sea levels after the last glacial maximum ∼6,500 years. As these meadows are geographically fragmented and unlikely to experience any contemporary gene flow via seed or pollen dispersal (Evans et al., 2014), the dominant clones existing within these meadows are assumed to be capable of withstanding local environmental changes by having highly plastic phenotypes. Indeed, our results show that different genotypes within meadows vary widely in their physiological and morphological response to reduced light (**Figures 2B, 3**). A change in growth rates as a plastic response to shading may initially assist in shoot survival, but may not necessarily benefit the plant in the long-term. Prolonged exposure to shading can induce photoacclimation to the lower light regime, which is associated with a drop in respiration rates, resulting in shorter, stunted growth forms and lower productivity (Collier et al., 2012). The rapidly reduced growth rates of *P. australis* in Lake Macquarie and Jervis Bay, suggests these vulnerable clonal plants are at risk of loss or damage under rapid environmental change (e.g., reduced light availability due to increased turbidity), regardless of the plasticity of existing genotypes.

The variation among seagrass genotypes in their tolerance to shading adds to the recent literature that quantifies the degree of intraspecific variation in tolerance to a range of environmental stressors in marine organisms (e.g., to ocean warming in macroalgae, Clark et al., 2013; to warming and acidification in sea urchins, Foo et al., 2014). Thus, complementarity via a greater variety of genotypes is still considered most beneficial in providing an ‘insurance’ effect against disturbance. We modeled how increased numbers of genotypes could be advantageous during environmental disturbance using the photosynthetic efficiency data (*F*_V_/*F*_M_), in which higher values represent greater acclimation to reduced light. Terrestrial research suggests that an optimal value of *F*_V_/*F*_M_ for over 70 different plants under non-stressed conditions is 0.83 (Björkman and Demmig, 1987), with values lower than this indicative of plant stress or possible damage to photosystem II (Roháček and Barták, 1999). Healthy seagrass plants may record values slightly lower than this (e.g., 0.75 for *Halophila ovalis*; Beer et al., 2014). As our study recorded values ranging from 0.68 to 0.81, we chose a ‘high-performance’ threshold of 0.8, with shoots obtaining values equal to or greater than this considered those best acclimated to low-light conditions.

Repeated resampling of the pooled photosynthetic performance data using varying numbers of genotypes in each sample revealed that there is a 90% or greater likelihood of including a high performing genotype when four or more genotypes are included within a meadow (i.e., those with photosynthetic efficiency values of 0.8 or greater; **Figure 4**). To go one step further, obtaining a 90% likelihood of including a genotype with an average *F*_V_/*F*_M_ of 0.81 or greater (the highest recorded value in this study) would require eight or more genotypes. It should be noted that this is a very simplified model of a theoretical meadow consisting of randomly selected genotypes from all those we sampled, although it does utilize realistic data from the 17 genotypes sampled *in situ*. Additionally, while photosynthetic efficiency as measured by *F*_V_/*F*_M_ is commonly used as a measure of plant stress and performance, it does represent only a snapshot of efficiency at the instant the measurement is made. Thus, it is important to note that assumptions are made when interpreting the overall ‘performance’ of plants based on *F*_V_/*F*_M_ data, and morphological measures such as growth are perhaps a more accurate representation of overall performance. Nonetheless, the results of the simulation support our original hypothesis that including more genotypes is always advantageous in terms of adaptive capacity, no matter how well-adapted one particular genotype is to the existing local conditions. This has significant implications for *P. australis* meadows that are almost entirely made up of only one to three genotypic clones, such as those found on the south-eastern coastline of Australia, close to the species range edge (Evans et al., 2014).

As individual genotypes that exhibit high photosynthetic performance under reduced light are increasingly likely to be found in meadows with four or more genotypic clones, we can hypothesize that under longer durations of reduced light (months to years), meadows with a high number of genotypes will also be better able to maintain high productivity. This is an obvious advantage, particularly given the unprecedented rate of environmental change experienced by seagrass meadows in the 21st century (Unsworth et al., 2014). An ever increasing human population size has the potential to further impact already vulnerable seagrasses in estuarine environments (Orth et al., 2006): coastal housing developments, land reclamation and commercial dredging have all been directly linked to seagrass decline from reduced light as a result of increased turbidity (Short et al., 1996; Lotze et al., 2006; Ralph et al., 2006; Walker et al., 2006; Yaakub et al., 2014). Past exposure to prolonged turbidity does not always predict future performance under additional stress, with seagrasses either performing better (Maxwell et al., 2014) or worse (Yaakub et al., 2014) than those from more ‘pristine’ meadows. It will also be important to investigate how stress due to changes in light availability may interact with additional stressors such as temperature (e.g., York et al., 2013).

With *P. australis* already listed as near-threatened on the IUCN Red List, effective management of future declines and impacts is a necessity, and must utilize information on individual meadow and genotype performance, particularly under stressful conditions. This research highlights the importance of incorporating genotypic information when investigating differences in plant stress response. While most *P. australis* meadows were generally resilient to short-term shading stress, variation in stress response is likely driven by the performance of individual genotypes. Although low diversity meadows may contain genotypes that perform well under stress, increasing the number of genotypes within a seagrass meadow greatly increases the likelihood of including a high performing genotype that will be more resilient to a future changes in light conditions. These results have significant implications for restoration and rehabilitation efforts, particularly for the sourcing of appropriate donor meadows and genotypes for transplantation.

## Author Contributions

SE, AV, and AP conceived and designed the study, SE performed the experiments, SE and AP analyzed the data. SE, AV, and AP wrote the paper.

## Conflict of Interest Statement

The authors declare that the research was conducted in the absence of any commercial or financial relationships that could be construed as a potential conflict of interest.
